# Molecular response of canine testis to GnRH agonist: Insights into AR, HIF-1α, and HSPs expression during arrest and recovery of spermatogenesis

**DOI:** 10.1016/j.cstres.2024.11.007

**Published:** 2024-12-02

**Authors:** Anastasiia Vasetska, Eva-Maria Packeiser, Hanna Körber, Selim Aslan, Serhan Ay, Murat Findik, Firdevs Binli, Murat Selçuk, Christelle Speiser-Fontaine, Sandra Goericke-Pesch

**Affiliations:** 1 Unit for Reproductive Medicine – Clinic for Small Animals, University of Veterinary Medicine Hannover, Foundation, Hannover, Germany; 2 Department of Obstetrics and Gynaecology – Faculty of Veterinary Medicine, Near East University, Nicosia, Cyprus; 3 Department of Obstetrics and Gynaecology – Faculty of Veterinary Medicine, Ondokuz Mayıs University, Samsun, Turkey; 4 Department of Reproduction and Artificial Insemination – Faculty of Veterinary Medicine, Ondokuz Mayıs University, Samsun, Turkey; 5 Companion Animal Medical Department, Virbac Group, Carros, France

**Keywords:** GnRH agonist slow-release implant, Androgen receptor, Dog testis, Heat shock protein, Cellular stress

## Abstract

Slow-release gonadotropin-releasing hormone (GnRH) agonist implants are frequently used for contraception in male dogs. Although the effects are fully reversible, there is still concern about the safety of the implant’s mode of action. Addressing this, we investigated cellular stress and androgen receptor (AR) signaling during downregulation and recovery. Testicular tissues were sampled from dogs castrated at different time points after GnRH implant removal and compared with untreated controls. *AR*, hypoxia-inducible factor 1 (*HIF1A*), heat shock proteins heat shock protein 72 (*HSP72*), heat shock protein 73 (heat shock cognate, HSPA8) (*HSP73*), heat shock protein A2 (*HSPA2*), heat shock protein 90 alpha (inducible isoform) (*HSP90AA1*), and heat shock protein 90 beta (constitutive isoform) (*HSP90AB1*) were investigated by quantitative real-time polymerase chain reaction and AR, HSP72, HSP73, and HSP90 immunohistochemically. While *AR*, *HIF1A*, and *HSP70* were upregulated at gene expression level, *HSPA8*, *HSPA2*, and *HSP90AA1* expression were downregulated during spermatogenic arrest; *HSP90AB1* expression did not change. Immunohistochemistry verified AR-expression in Sertoli, peritubular, and Leydig cells, occasionally also in spermatogonia. Stress-inducible HSP72 was occasionally detected, while constitutive HSP73 and HSP90 were abundantly expressed by germ cells. Our results were similar to studies on seasonal breeders such as pine voles, geese, fish, and soft-shelled turtles. Accordingly, GnRH implants did not impose additional cellular stress on testicular cells when compared with natural recrudescence. Since comparative data on HIF1α are scarce, we cannot draw conclusions about hypoxic conditions.

## Introduction

Gonadotropin-releasing hormone (GnRH) agonist implants are well known in veterinary practice to induce temporary infertility in male dogs and that is what they are frequently used for since their registration in Australia in 2004 and in Europe in 2007. While surgical castration results in removal of the source of testosterone and rendering the dog permanently infertile, GnRH agonist implants provide a relatively convenient way of managing fertility (and androgen-related behavior and diseases) that can nevertheless be reversed when the implant is withdrawn or when the efficacy of the implant has waned. This makes the GnRH agonist implants suitable for those owners who want to keep the decision on reproductive control open without having to perform an invasive, permanent, and irreversible surgery.^1^, ^2^ In human medicine, these implants are applied to delay early puberty in children^3^ and to treat androgen-dependent prostate cancer.^4^, ^5^, ^6^ Interestingly, GnRH slow release implants even improved cognitive function in patients with down syndrome.^7^

Spermatogenesis is hormonally regulated. In a physiologic state, endogenous GnRH is secreted by the hypothalamus in pulses to stimulate the anterior pituitary gland to secrete luteinizing hormone (LH) and follicle-stimulating hormone (FSH), which induce testosterone synthesis and spermatogenesis in the testis. However, GnRH agonist implants interfere with this process through continuous release of synthetic GnRH analogs: This causes an initial increase in LH and FSH production—the so-called “flare-up effect”—and subsequently inhibits and downregulates the pituitary GnRH receptors. This in turn desensitizes the hypothalamic pituitary axis, suppresses the pulsatile release of GnRH, and triggers a downregulation of LH and FSH secretion. Consequently, the testicular testosterone synthesis, the final effector in the cascade, decreases, which together with the decrease in FSH finally interrupts the spermatogenic process.^8^, ^9^ In seasonal breeders, environmental factors such as daylight, temperature, and food availability naturally influence the pulsatile hypothalamic GnRH release.^10^ During sexual inactivity, testicular testosterone production and spermatogenesis ceases, the testis volume shrinks, and testicular androgen receptor (AR) expression adapts,^11^, ^12^, ^13^, ^14^, ^15^ similar to male dogs treated with GnRH agonists.^16^, ^17^

AR mediates physiological and pathophysiological effects of androgens, including spermatogenesis, prostate development, and cancer progression by binding androgen response elements, which influence transcription of AR target genes.^18^, ^19^, ^20^ In the testis AR mainly localizes to Sertoli cells, Leydig cells, and peritubular cells,^21^ also in the dog.^16^

Hypoxia-induced factor (HIF)-1 is another significant factor for the maintenance of undisturbed spermatogenesis. Both upregulation and downregulation in response to a changed oxygen supply in the tissue negatively influence spermatogenesis.^22^ The expression of the herein investigated a subunit (*HIF1A*) is inducible.^23^

Heat shock proteins (HSPs) are a large group of chaperones, endogenous protective proteins that are located in the cytoplasm and nucleus to maintain normal cellular function. HSPs are able to suppress proinflammatory cytokines, reduce oxidative stress, repair ion channels, protect against different toxic effects, modulate immune-mediated injures and prevent apoptosis.^24^ These proteins also assist specific client proteins such as HIF-1 and the inactive, unbound AR in folding, structural stabilization, and conservation.^23^, ^25^ Other functions are protein transport, modification, and secretion.^26^, ^27^ According to their molecular weights of 27, 40, 60, 70, and 90 kDa, HSPs are assigned to the correspondingly named families. While some isoforms are inducible by cellular stress, other isoforms are constitutively expressed. HSP70 can be further differentiated into the stress-induced 72 kDa-form HSP72 (*HSP70*) and the constitutively expressed HSP73 (heat shock cognate HSC70; *HSPA8*).^28^ Likely, HSP90 appears in two main mammalian isoforms with inducible HSP90α (*HSPAA1*) and constitutively expressed HSP90β (*HSPAB1*).^29^ HSP70 family member HSPA2 expression is enriched in the testis and inducible by HIF-1 transcription factor.^30^ Although being crucial for spermatogenesis, the exact role of HSPA2 in spermatogenesis is poorly investigated so far.

Although first spermatozoa were detected 7–12 weeks and semen quality restoration was observed 12–19 weeks after the GnRH agonist implant removal^31^, ^32^ with individual variation, there is little information at the molecular level regarding cellular stress and AR signaling. By monitoring AR, HIF-1, AR, and their chaperones HSP72, HSP73 HSP90α, and HSP90β, as well as HSPA2 from the state of downregulated spermatogenesis until full recovery, we aimed to contribute to a characterization of HSP function in dogs and evaluated whether the level of cellular stress is comparable with natural regulation of sexual activity in natural seasonal breeders.

## Results

### AR expression

**Fig. 1:**
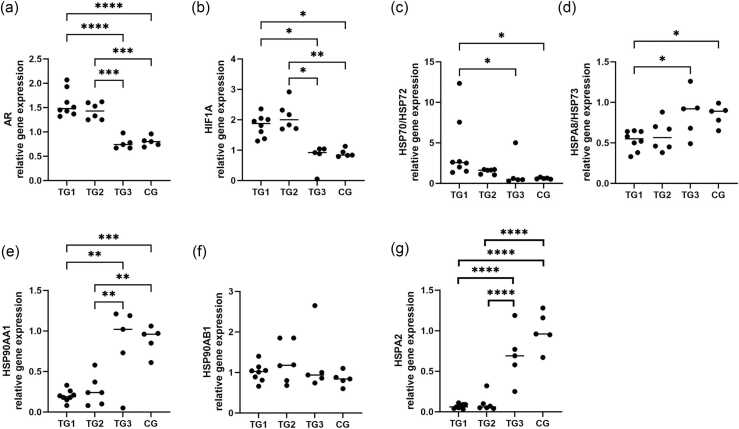
Relative gene expression of *AR* (a), *HIF1A* (b), *HSP70* (HSP72) (c), *HSPA8* (HSP73) (d), *HSP90AA1* (e), *HSP90AB1* (f), and *HSPA2* (g) in canine testis, groups of downregulation (TG1), early recovery (TG2), late recovery (TG3), and healthy control dogs (CG).

The messenger ribonucleic acid (mRNA) expression exhibited significant differences between treated groups (TGs) and control group (CG) based on analysis of variance (ANOVA) (*P* < 0.0001). The *AR* mRNA expression was significantly higher in the downregulated spermatogenesis period compared to recovery and control (Figure 1(a); TG1 vs. TG3 *P* < 0.0001, TG1 vs. CG *P* < 0.0001, TG2 vs. TG3 *P* = 0.0001, TG2 vs. CG *P* = 0.0002).

**Fig. 2:**
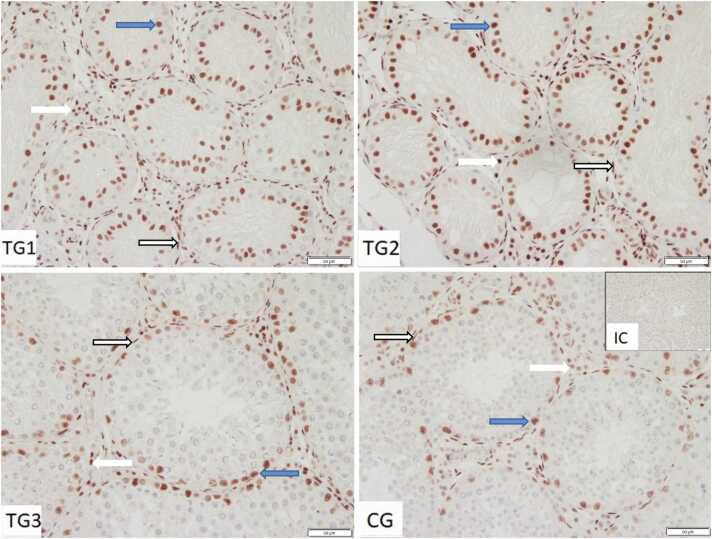
Immunostaining against androgen receptor (AR) in control group (CG) and Treated groups of dogs (TG1, TG2, TG3) (200×magnification). IC isotype control. Sertoli cells (*blue arrow),* Leydig cells (*white bold arrow*), peritubular cells (*white outlined arrow*).

Specific immunopositive signals against AR protein were observed in the nuclei of Sertoli cells, as well as in Leydig cells and peritubular cells (Figure 2). Occasionally, some spermatogonia stained weakly immunopositive.

**Fig. 3:**
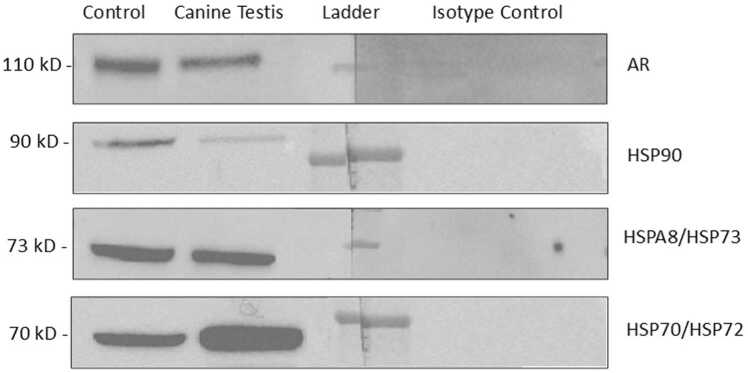
Western Blot analysis of androgen receptor (AR), HSP90, HSPA8/HSP73, HSP70/HSP72; Canine Testis: adult, healthy canine testis tissue; Control: positive control (mouse testis tissue for AR, heat-shocked HeLa cell lysate for HSPs); isotype control.

Western blots were performed to confirm the specific reactivity of the antibodies used in immunohistochemistry (IHC). A single band, observed at 110 kD in both, canine testicular tissue and homogenate from mouse testicular tissue as positive control, demonstrated specific protein reactivity (Figure 3 AR). No specific or unspecific band was visible in the negative and isotype controls.

### Hypoxia-induced factor 1 (HIF1A) expression

Similarly to *AR*, *HIF1A* expression differed significantly between the groups (Kruskal-Wallis test *P* = 0.0006, Figure 1(b)). TG1 and TG2 testes upregulated *HIF1A* compared to TG3 (*P* = 0.0476 and *P* = 0.0140) and compared to CG (*P* = 0.0303 and *P* = 0.0087). There were no significant differences in *HIF1A* expression between TG1 and TG2 or between TG3 and CG.

### Expression of the stress-induced heat shock protein 72 (HSP70)

The mRNA expression for *HSP70* (HSP72) differed significantly (Kruskal–Wallis-test *P* = 0.0042), with significantly higher expression in TG1 compared to TG3 (*P* = 0.0213) and CG (*P* = 0.0131) (Figure 1(c)).

**Fig. 4:**
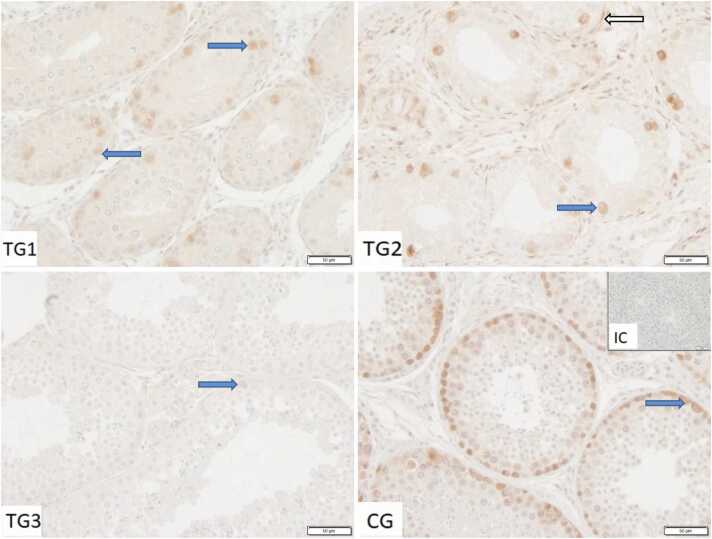
Immunostaining against HSP70/HSP72 in control group (CG) and treated groups of dogs (TG1, TG2, TG3) (200×magnification). IC isotype control. Sertoli cells (*blue arrow),* peritubular cells (*white outlined arrow*).

In all groups, very few Sertoli cells stained positive for HSP72 (Figure 4). The observed staining occurred in the perinuclear cytoplasm, not ranging toward the adluminal compartment of the cell. Additionally, individual peritubular cells, few spermatogonia (nuclear and cytoplasmic), as well as very few cells in the interstitial compartment, most likely immune cells, stained positive.

The Western blot for HSP70 revealed a specific immunoreactive band using protein homogenate from adult canine testis and heat shocked HeLa cell lysate as a positive control (Figure 3 HSP70/HSP72). The molecular weight of the canine testis band was located at the predicted molecular weight of approximately 70 kD, indicating specificity.

### Expression of the constitutive heat shock protein 73 (HSPA8)

Contrarily to the beforementioned genes, *HSPA8* (HSP73) was significantly downregulated in suppressed spermatogenesis (ANOVA, *P* = 0.0099; TG1 vs. TG3 *P* = 0.0284; TG1 vs. CG *P* = 0.0375).

**Fig. 5:**
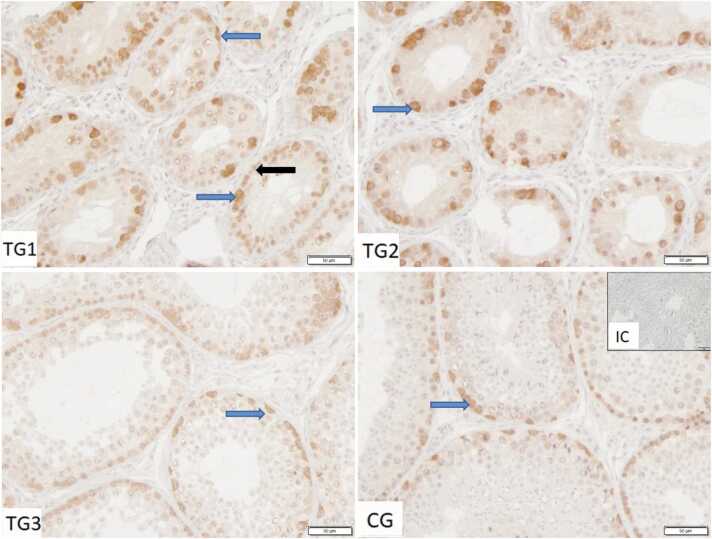
Immunostaining against HSP73 in control group and treated groups of dogs (TG1, TG2, TG3) (200×magnification). IC isotype control. Sertoli cells (*blue arrow),* germ cells (*black bold arrow*).

IHC revealed positive specific staining against HSP73 regularly in Sertoli cells, but not in all (Figure 5). Comparably to HSP72, the staining was located in the perinuclear cytoplasm, close to the basal membrane. Additionally, solitary germ cells showed diffuse cytoplasmic staining.

The Western blot for HSP73 revealed a specific immunoreactive band using protein homogenate from adult dog testis and HeLa heat shock cell line as recommended positive controls. The molecular weight of the canine band was located within the predicted range of 66–78 kD, specifically at approximately 73 kD (Figure 3 HSPA8/HSP73).

### Heat shock protein 90 expression (inducible HSP90AA1; constitutive HSP90AB1)

ANOVA revealed significant differences between the groups for *HSPAA1* expression (*P* < 0.0001, Figure 1(e)). Similar to *HSPA8*, *HSP90AA1* mRNA levels in late recovery and CG testes were higher than in the downregulated and early recovery testes (TG1 vs. TG3 *P* = 0.0012, TG1 vs. CG *P* = 0.0005, TG2 vs. TG3 *P* = 0.0062, and TG2 vs. CG *P* = 0.0029). *HSP90AB1* mRNA ratios did not differ significantly between the groups (Figure 1(g)).

**Fig. 6:**
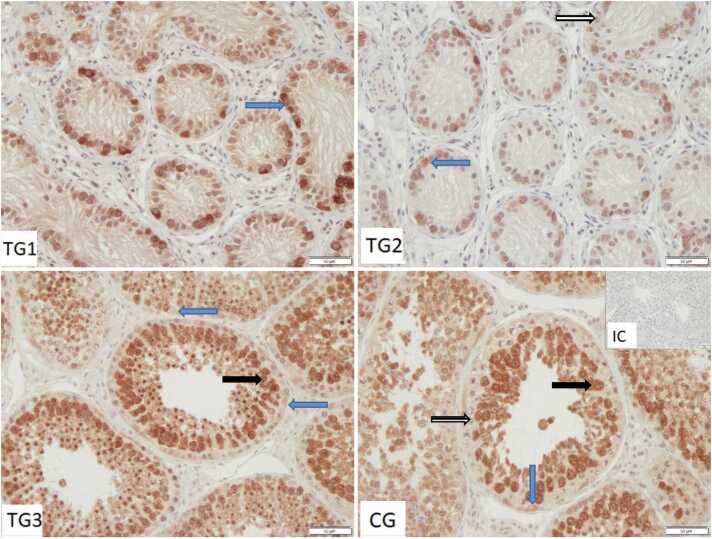
Immunostaining against HSP90 in control group and treated groups dogs (TG1, TG2, TG3) (200×magnification). IC isotype control. Sertoli cells (*blue arrow),* spermatogonia *(striped arrow),* immunopositive germ cells (*black bold arrow*).

Immunopositive staining against HSP90 was specifically observed in the cytoplasm of germ cells (Figure 6). The staining appeared to be stage-specific (TG3 and CG, Figure 6), with spermatogonia and early spermatocytes exhibiting a weak granulated staining pattern, whereas spermatocytes in the late pachytene and round spermatids showing a particularly intense staining. The staining intensity decreased in elongating spermatids and further in elongated spermatids along with the reduction of cytoplasmic volume. Consequently, in TG1 and TG2 only weak staining was observed, as spermatogenesis was arrested at spermatogonia and early spermatocytes. Sertoli cells were predominantly negative, sometimes weakly stained.

The specificity and reactivity of the HSP90 antibody were confirmed through Western blot analysis using canine testicular tissue and an appropriate positive control. The canine testis band, observed at approximately 90 kD, corresponded to the molecular weight of the positive control (Figure 3 HSP90).

### Testis-enriched heat shock protein A2 expression (HSPA2)

Similarly to *HSP90AA1*, *HSPA2* mRNA abundances differed significantly (ANOVA of log-transformed data, *P* < 0.0001, Figure 1(f)) with higher expression in late recovery and CG (TG1 vs. TG3, TG1 vs. CG, TG2 vs. TG3, and TG2 vs. CG, all *P* < 0.0001).

## Discussion

For years, GnRH agonist slow release implants are frequently used for temporary downregulation and contraception in male dogs, and thus their clinical and functional effects are intensively studied at this point.^17^, ^31^, ^32^, ^33^, ^34^, ^35^ However, information on the induced changes at molecular level in the testis is scarce. With the present study, we monitored the expression of AR, HIF-1, and their HSP chaperones during normal spermatogenesis, downregulation, and recovery.

AR staining was observed in Sertoli cells, Leydig cells, and peritubular cells, as expected and previously shown^16^ whereas most germ cells were AR-negative. AR expression in germ cells is controversial and potentially species-dependent,^36^ with sporadic AR expression in spermatogonia in dogs in another study,^16^ too. As in other IHC studies, AR staining was nuclear despite the inactive form of the receptor is located in the cytoplasm and first translocates to the nucleus if bound to testosterone. As previously stated, inactive AR is complexed by HSPs in the cytoplasm. Possibly the HSPs occupy and therefore mask the antigens of the AR protein preventing cytoplasmic binding in IHC. Studies analyzing the translocation process itself use fluorescent AR fusion proteins for localization, making cytoplasmic AR visible.^37^ The noted AR upregulation at mRNA level in downregulated spermatogenesis is in accordance with observations made during sexual inactivity in seasonal breeders like hibernating fish (*Onychostoma macrolepis*),^38^ geese,^39^ and pine voles.^40^ The authors discussed that phenomenon as a response to lower testosterone levels and a status of increased sensitivity and readiness for a rapid return to full spermatogenesis toward the beginning of the next breeding season.^38^ Nevertheless, in our dogs, as well as in humans, mice, cattle, bats, pigs, and horses,^8^, ^21^, ^41^, ^42^, ^43^, ^44^, ^45^, ^46^, ^47^ AR is expressed in all cell types but germ cells (with very few exceptionally stained spermatogonia in our study). With downregulated spermatogenesis, the percentage of germ cells and therewith the tubulus/interstitium ratio and the testis volume decrease,^16^ which was also confirmed by all aforementioned studies of seasonal breeders. Consequently, the increased expression of AR might be due to relative enrichment of AR-expressing cells. With regard to the Sertoli or Leydig cells, AR-upregulation might be less prominent than proposed by gene expression levels of whole tissue lysates. Different to dogs, in the bat, AR protein expression during sexual inactivity does not differ from breeding season but is prominently upregulated during recovery.^48^ In wild ground squirrels, however, the situation is inverse,^49^ meaning AR mRNA and protein are upregulated only during the breeding season. In contrast to our results with *AR* mRNA upregulation during suppressed spermatogenesis, we observed less AR-immunopositive Sertoli cells in a previous study.^16^ Additionally and contrary to the present and previous results using *in-situ* hybridization,^16^ *AR* mRNA was solely located in Sertoli cells. In conclusion, AR regulation appears to be species-specific and special care should be taken while interpreting the results obtained with different methodologies.

Different to other stem cell pools, the spermatogonial stem cell niche is adapted to low oxygen supply,^50^, ^51^ as the capillary network of the testis is relatively sparse and oxygen supply even decreases toward the tubular lumen. As part of an adaption to these conditions, HIF1α plays an important role in regulating the expression of testis-specific variants of glycolytic enzymes. In humans and mice, even a switch from ubiquitous HIF1α expression to a testis-specific isoform was described, regulated by two different promoter regions.^51^ If a similar mechanism exists for the dog, is still unknown. The human protein atlas locates testicular HIF1α expression exclusively in germ cells.^52^ Considering the beforementioned decreased ratio of tubulus/interstitium volume and the reduced number of germ cells in our downregulated testis tissues, the observed *HIF1A* upregulation is even more prominent and indicates a significant role during downregulated spermatogenesis and early recovery. HIF1α was upregulated in a rat varicocele model, a hypoxic condition associated with increased apoptosis.^22^ However, HIF1α is a transcription factor inducing both, proapoptotic and antiapoptotic effects.^53^ HSP90α knockout mice showed a normal phenotype, besides complete failure of spermatogenesis.^54^ A closer look at the mechanism using *in vitro* assays revealed a concomitant decrease of HIF1α protein in testis tissue and cell lines.^23^ The authors concluded that HSP90α complexes and protects HIF1α protein from degradation in order to maintain its specific role in the hypoxic testicular environment. Since *HSP90AA1* (expressed in human Sertoli and germ cells) is downregulated in TG1 and TG2, this protection of HIF1α protein is possibly reduced. If the upregulation of *HIF1A* mRNA is compensatory to HIF1α protein degradation, or due to other underlying mechanisms during spermatogenesis, remains to be investigated. Nevertheless, the described situation remains until normal spermatogenesis is histologically confirmed in TG3 and CG, which supports the connection of HIF1α and HSP90α. In our samples, the hormonal stimulus for spermatogenesis is suppressed and we have no hint toward a changed tissue oxygenation. Contrarily, in case of varicocele in humans, malformation of the spermatic veins induces oxidative stress, hypoxia and hyperthermia in the testis, leading to decreased sperm quality and apoptosis.^22^ Interestingly, the suppression of elevated testicular HIF1α expression to normal levels improved the negative effects in the rat varicocele model. In case of disease, oxidative stress, and possibly infection, excessive HIF1α expression might on the other hand promote germ cell apoptosis. Taken together, the regulation of HIF1α and cofactors appear to be complex, with a certain expression level being required for functional spermatogenesis and either upregulation or downregulation being disadvantageous.

As demonstrated in humans, *HSPA2* is remarkably higher expressed in the testis, compared to other organs, underlining its special role in spermatogenesis. A study conducted in Chinese soft-shelled turtles not only verified this observation but also detected an upregulation of testicular HSPA2 mRNA and protein expression in the breeding season,^55^ which is in good agreement with our observations. HSP73/HSPA8 showed a similar expression pattern in our dogs, as well as in the Chinese soft-shelled turtles, both at mRNA and protein level. In their transcriptomic analysis, the authors associated both genes with MAPK signaling, a fundamental pathway participating in cellular stress response and apoptosis regulation.^55^ Apoptosis and proliferation of germ cells is regulated and balanced in times of downregulated and upregulated spermatogenesis.^56^ With a previous study,^56^ the authors were able to link elevated *HSPA2* and *HSPA8* levels with apoptosis prevention during active spermatogenesis in the breeding season. This might most likely might be applicable to artificial downregulation of spermatogenesis by GnRH slow release implants in dogs, as well.

In contrast to HSPA2 and HSP73, HSP72 was slightly upregulated in our samples of arrested spermatogenesis, both at mRNA and protein level. Nevertheless, only few Sertoli and germ cells stained positive for HSP72, an HSP which is actively expressed in condition of cellular stress.^57^ Additionally, HSP72 was detected in all groups, indicating that the stage of arrested spermatogenesis, as well as the process of recovery, do not impose cellular stress on the canine testis. In contrast, experimentally applied thermal stress significantly upregulated HSP72 in rabbit testes. In the Chinese soft-shelled turtles, upregulation was observed toward the end of the breeding season.^55^ Since we did not monitor HSP72 expression during downregulation directly after implantation, we cannot exclude cellular stress in the testis during this phase.

HSP90 was located in the cytoplasm, as described for human HSP90.^29^ The cytoplasmic localization of all investigated HSPs in germ cells, as well as partly in Sertoli cells is in accordance with observations in other species, as reviewed by Huang *et al.*^58^; however, Leydig cells did not stain in our study. More specifically, the exclusively perinuclear immunopositive signal in the Sertoli cells, especially for HSP72 and HSP73 underlines the function of HSPs in translocating AR to the nucleus,^59^ as testosterone enters the tubulus from the basal membrane. In stressless conditions, only 5–10% of HSP90 is located in the nucleus,^29^ again indicating that our GnRH slow release implants do not induce cellular stress. Due to the different gene expression results for the two HSP90 isoforms, with HSP90AA1 being downregulated in suppressed spermatogenesis and HSP90AB1 being indifferently expressed, a closer look at protein localization is reasonable. Our used antibody recognizes both isoforms and mostly represents the picture of human HSP90α as depicted by the human protein atlas (https://www.proteinatlas.org/ version 23.0),^60^, ^61^ with abundant and stage-specific expression in germ cells. Corresponding to our classification of groups, TG3 and CG possess full spermatogenesis, which in turn explains the higher *HSP90AA1* gene expression. *HSP90AB1* however is expressed in Sertoli cells and spermatogonia in human testis (https://www.proteinatlas.org/ version 23.0).^60^, ^61^ Both cell types do not seem to change in number during suppressed spermatogenesis in our samples. Due to the beforementioned shift in the germ cells/Sertoli cells proportion, our observed unchanged gene expression in whole tissue lysates propose an *HSP90AB1* upregulation in Sertoli cells and spermatogonia. The differing expression of both HSP90 isoforms, as well as the stage-specific IHC staining pattern however indicate isotype-specific task assignment in germ cells in dogs, which might be worth further investigation. Although both isoforms demonstrate similar client protein interactions, HSP90α expression is described as stress-inducible and might be important in infections and hypoxia, while HSP90β is stably expressed.^62^ Its use as a reference gene for gene expression studies as occasionally done^63^ is however not recommended for the testis based on our results, given the stage-specific expression and the variations especially in TG3. Interestingly, Grad *et al.* discovered elevated HSP90α expression in comparison with other tissues in mice, indicating a testis-specific role for the alpha isoform. Moreover, while HSP90β knockout mice embryos die prior to implantation, HSP90α mutant mice are vital except for disrupted spermatogenesis beyond pachytene.^54^ Grad *et al.*’s HSP90α-deficient mice however showed normal Sertoli cell number and function,^54^ indicating a predominant role of HSP90β in Sertoli cells, complexing, folding and stabilizing AR and other client proteins. HSP90α on the other hand secures germ cell proliferation and differentiation, presumably by stabilizing and activating HIF1 and the testis specific serin/threonine kinases^23^, ^64^ and thereby preventing mitotic arrest and apoptosis.^54^ In boars, HSP90β was detected in prepubertal and postpubertal testes, while HSP90α was absent in immature samples.^58^ In other words, HSP90α’s role seems to be restricted to germ cell proliferation and differentiation and gets lost during epididymal passage.^65^ The specific roles of the two HSP90 isoforms in dogs, however, warrant further investigation.

## Conclusion

In conclusion, our study confirms the important roles of AR, HIF-1, HSPA2, and HSP90α in regulating spermatogenesis. Our findings indicate that artificial downregulation of spermatogenesis by GnRH slow release implants in dogs seems to be comparable with natural testicular inactivity in seasonal breeders. Importantly, the state of arrested spermatogenesis, as well as the process of recovery, do not seem to apply cellular stress to the testis. However, due to the study design, we cannot make any statement about the process of downregulation itself. An upregulation of HIF1α might indicate hypoxic conditions; it might however as well be reactive to increased protein turnover. Since Sertoli cells appeared to be the only cell type, in which all applied methodologies reliably detected AR, and which additionally expressed HSPs and HIF1α, they might be a particularly suitable cell type to further investigate the mechanism of HSP-AR complexation, AR signaling and AR transport in the canine testis. Canine Sertoli cell cultures might be a useful *in vitro* model for a closer look at molecular and functional level.

## Materials and methods

### Sample collection

Animal experimentation was approved by the Animal Experiments Local Ethics Committee of Ondokuz Mayis University (approval number: 2015/52).

Twenty-one clinically healthy, sexually mature adult male dogs of various breeds and crossbreeds, aged 18.4 ± 5.7 months (range: 12–48 months) with a mean body weight of 21.7 ± 5.4 kg (range: 11–40 kg) (TG) were implanted subcutaneously at the periumbilical area with a 4.7 mg deslorelin slow-release GnRH-agonist implant (Suprelorin®, 4.7 mg; Virbac, France). Five untreated crossbreed dogs aged 17.6 ± 4.27 months with a mean body weight of 20.2 ± 3.12 kg served as controls (CG). After 5 months of treatment the implant was removed under local anesthesia in all TG dogs. Randomly assigned to subgroups, the TG dogs were surgically castrated at the day of removal, or 1, 2, 3, 4, 5, 6, 7, 8, and 10 weeks afterwards and testis tissue was collected, preserved and prepared immediately after surgery as described previously.^31^, ^32^ For mRNA analysis, samples were preserved with RNAlater® (Ambion Biotechnologie GmbH, Wiesbaden, Germany) and stored at −80 °C. Samples subjected to histological and IHC analysis were fixed in Bouin’s solution followed by routine processing, embedding in paraffin wax and mounting onto blocks. Samples of CG dogs were processed accordingly.

Grouping of the TG dogs was based on testosterone concentrations and stage of spermatogenesis: TG1 (downregulation)—with testosterone ≤0.1 ng/ml, presenting arrest on the level of spermatogonia & spermatocytes in histological evaluation; TG2 (early recovery)—testosterone >0.1 ng/ml, arrest on the level of spermatogonia and spermatocytes; TG3 (late recovery)—presenting testosterone within reference ranges and physiological spermatogenesis.

### Isolation of total RNA, cDNA synthesis, and quantitative real-time PCR

For total RNA isolation, a Trizol-based protocol including a TissueLyzer LT (Qiagen, Germantown, MD, USA) was followed as previously described.^66^, ^67^ Concentrations of extracted RNA were measured photometrically using an IMPLEN NanoPhotometer® (IMPLEN GmbH, Munich, Germany). Due to insufficient RNA quality, one sample in TG1 and two samples in TG2 had to be excluded from the gene expression analysis. According to the manufacturer's instructions, reverse transcription of full-length first-strand cDNA was performed using DNase-pretreated RNA (200 ng/µl) and M-MLV Reverse Transcriptase, RNase H Minus (Promega Corporation, Madison, WI, USA) with RNasin® Plus RNase Inhibitor (Promega Corporation, Madison, WI, USA) and Random Primers (Promega Corporation, Madison, WI, USA).

The primers for the quantitative real-time polymerase chain reaction of *AR* were purchased from Kaneka Eurogentec S.A. Seraing, Belgium and primer sets for the *HSP* genes, designed with BLAST^68^ as well as primers for the reference genes glyceraldehyde-3-phosphate dehydrogenase (*GAPDH*), hypoxanthine guanine phosphoribosyltransferase (*HPRT*) and beta actin (*ACTB*) were from Microsynth (Microsynth AG; Balgach, Switzerland) (Table 1).

**Table 1:** Sequence of primers for RT-qPCR, amplicon length, efficiency, and accession number.

Primer	Accession Nr.		Sequence (5′→3′)	Amplicon	Efficiency
AR	XM_038450180.1	for	GAGCCAGGCGTGGTGTGT	69 bp	2.05
		rev	GCTAGAGAGCAAGGCGTCAAA		
HIF1A	NM_001287163.1	for	TTACGTTCCTTCGATCAGTTGTCA	106 bp	2.07
		rev	GAGGAGGTTCTTGCATTGGAGTC		
HSP70/HSP72	NM_001003067.3	for	CTCCACCCGTATCCCCAAGG	70 bp	2.11
		rev	GCTCTTGTTGAGATCGCGGC		
HSPA8/HSP73	XM_038664592.1	for	GGAAGGTTCTGAGGCAGGGTA	142 bp	2.08
		rev	CAGGTCCCTTAGACATAATTGCTTC		
HSP90AA1	XM_038545759.1	for	CTTGACCGATCCCAGTAAGC	128 bp	2.09
		rev	TATTGATCAGGTCGGCCTTC		
HSP90AB1	XM_038683654.1	for	CCTCGTCGGGCTCCTTTTGA	76 bp	2.12
		rev	ACACACGGCGGACATACAGT		
HSPA2	XM_038544921.1	for	CGAGGGAGGGAAACCCAAGG	161 bp	2.10
		rev	AGTAAGCCGGGACCGTGATG		
HPRT	NM_001003357.2	for	TGACACTGGGAAAACAATGCA	94 bp	2.10
		rev	GGTCCTTTTCACCAGCAAGCT		
GAPDH	NM_001003142	for	GGCCAAGAGGGTCATCATCTC	228 bp	2.10
		rev	GGGGCCGTCCACGGTCTTCT		
ACTB	NM_001195845.3	for	GCTGTGCTGTCCCTGTATG	97 bp	2.17
		rev	GCGTACCCCTCATAGATGG		

Abbreviations used: for, forward; rev, reverse; RT-qPCR, quantitative real-time polymerase chain reaction.

For quantitative real-time PCR, we used a Roche Diagnostics GmbH LightCycler® 96 real-time PCR system (Software version 1.1.0.1320) and ran all samples in triplicates according to our previously published protocol.^69^ RNase-free water served as non-template control. PCR efficiencies for all primers (Table 1) were calculated using standard curves from a cDNA dilution series in triplicates (1:4, 1:8, 1:16: 1:32, 1:64, 1:128). Specific primer binding was confirmed by sequencing the amplicons (Microsynth AG). Gene expression ratios were calculated normalized to the two most stably expressed reference genes *HPRT* and *GAPDH* and taking into account the respective primer efficiencies.^70^

### Immunohistochemistry

Localization and protein expression patterns in canine testicular samples were analyzed for AR, HSP70/72, HSC/HSP73 and HSP 90 by IHC. Bouin-fixed, paraffin-embedded testicular tissue slides (3 µm) were mounted on SuperFrost-Plus slides (Menzel Glaeser, Braunschweig, Germany), deparaffinized in xylene and rehydrated in graded ethanol dilution series, before antigen demasking by pretreatment with cooking citrate buffer (pH = 6). Endogenous peroxidase activity was inhibited by 3% hydrogen peroxide in methanol. To block unspecific binding sites, 10% goat serum and 5% BSA in ICC buffer (6,74 mM Na_2_HPO_4_, 1,47 mM KH_2_PO_4_, 2,68 mM KCl, 136,89 mM NaCl, 0,3% Triton, pH = 7.3), was used for AR and HSC/HSP73, but 10% horse serum and 5% BSA in ICC buffer for HSP90 and HSP70/72, followed by an overnight incubation at 4 °C with the AR, HSP 90, HSP70/72, HSC/HSP73 primary antibody (Table 2). Each step was followed by ICC buffer washings. Host-matched biotinylated secondary antibodies (Table 2), diluted 1:200 in blocking buffer, were applied for 30 min and the signals were visualized using an immunoperoxidase system (VECTASTAIN PK-6101 Rabbit IgG Elite ABC Kit and Vector Nova-RED Substrate Kit SK-4800, Vector Laboratories, Newark, CA, USA). For negative and isotype controls, ICC buffer was used, or the primary antibodies were replaced by host-matched IgG irrelevant isotype controls (I-2000 Mouse IgG Control Antibody from Vector Laboratories, clone RTK4530 Purified Rat IgG2b from BioLegend and I-1000 Rabbit IgG Control Antibody from Vector Laboratories).

**Table 2:** Antibodies used for IHC and Western blot for the detection of androgen receptor (AR) and heat shock protein 72, 73, and 90 (HSP70/72, HSC/HSP73, HSP90) in canine testicular tissues.

Target	Company	Catalog number	Host; clonality	Concen-tration (dilution) IHC	Secondary antibody IHC & Western blot	
AR	Abcam	ab227678	Rabbit monoclonal	9.4 µg/ml (1:50)	BA-1000^a^	PI-1000^a^
HSP72	Enzo Life Sciences	ADI-SPA-810	Mouse monoclonal	25 µg/ml (1:40)	BA-2000^a^	PI-2000^a^
HSP73	Enzo Life Sciences	ADI-SPA-815	Rat monoclonal	2 µg/ml (1:500)	BA-9400^a^	A18865^b^
HSP90	Enzo Life Sciences	ADI-SPA-830	Mouse monoclonal	2 µg/ml (1:500)	BA-2000^a^	PI-2000^a^

Abbreviation used: IHC, immunohistochemistry.

a Vector Laboratories.

b Thermo Fisher Scientific, Fremont, CA, USA.

The staining was evaluated visually under a light microscope (Olympus Bx 45, Olympus Europa SE & Co. KG, Hamburg, Germany) by two blinded investigators. An evaluation of the localization of immunopositive signals was conducted descriptively.

### Western blot

Western Blotting was used to test the protein expression of AR, HSP70/72, HSC/HSP73 and HSP 90 in canine testicular tissue and confirm specific binding of the used primary antibodies. Canine testis tissue lysate and corresponding positive controls (heat-shocked HeLa cell line for the HSPs and mouse testis tissue lysate for AR) were denatured at 95 °C for 10 min in Laemmli sample buffer in a water bath. On 4–20% gradient Mini-Protean® TGX™ Gels (Bio-Rad Laboratories, Hercules, CA, USA), we separated proteins by a sodium dodecyl sulfate-polyacrylamide gel electrophoresis. The proteins were blotted onto nitrocellulose membranes using a blotting system (Trans-Blot® TurboTM Transfer System, Bio-Rad Laboratories) and unspecific binding sites were blocked for five minutes at room temperature with EveryBlot Blocking Buffer (Bio-Rad Laboratories), diluted 1:1 in TBST (Tris buffered saline, 0.05% Tween 20). Afterwards, membranes were incubated overnight with the respective primary antibodies (Table 2), diluted in blocking buffer, at 4 °C, followed by three washes in TBST and incubation with HRP-conjugated secondary antibodies (Table 2) for one hour. ChemiDocTM Imaging Systems with Image LabTM Touch Software (Image Lab 6.0.1, Bio-Rad Laboratories) was used to visualize the signals using ClarityTM Western ECL Blotting Substrate (Bio-Rad Laboratories).

### Statistical Analysis

Statistical analysis of the qPCR data was performed using Microsoft Excel 2016 (Microsoft Corporation, Redmond, WA, USA) and Graph Pad Prism9 software (GraphPad Software, Inc., La Jolla, CA, USA). As normal distribution was confirmed for *AR*, *HSPA8*, *HSP90AA1* and log-transformed *HSPA2* data by Shapiro-Wilk test, ANOVA was applied, followed by Tukey’s post-hoc test to detect significances between the four treatment groups. Since *HIF1A*, *HSP70* and *HSP90AB1* were not normally distributed, neither untransformed, nor transformed, groups were compared using the Kruskal-Wallis test followed by Dunn’s multiple comparisons test. Statistical significance was defined for p<0.05. For comparative reason, data was displayed as scatter plots with median.

## Author contributions

**Hanna Körber:** Writing – review & editing, Validation, Investigation. **Eva-Maria Packeiser:** Writing – original draft, Visualization, Validation, Investigation, Formal analysis. **Anastasiia Vasetska:** Writing – original draft, Visualization, Validation, Investigation, Formal analysis. **Sandra Goericke-Pesch:** Writing – review & editing, Validation, Supervision, Resources, Project administration, Methodology, Formal analysis, Conceptualization. **Christelle Speiser-Fontaine:** Writing – review & editing, Resources, Conceptualization. **Murat Selçuk:** Writing – review & editing, Resources, Investigation. **Firdevs Binli:** Writing – review & editing, Resources, Investigation. **Murat Findik:** Writing – review & editing, Resources, Investigation. **Serhan Ay:** Writing – review & editing, Resources, Investigation. **Selim Aslan:** Writing – review & editing, Resources, Investigation.

## Declarations of interest

The authors declare the following financial interests/personal relationships which may be considered as potential competing interests: Sandra Goericke-Pesch reports financial support was provided by Virbac Ltd. Christelle Speiser Fontaine reports financial support was provided by Virbac Ltd. Goericke-Pesch reports a relationship with Virbac Ltd that includes: funding grants, speaking and lecture fees, and travel reimbursement. Christelle Speiser Fontaine reports a relationship with Virbac Ltd that includes: employment. Christelle Speiser-Fontaine is employed by VIRBAC, Companion Animal Medical Department, Carros, France, which also funded this study. SGP lectured for Virbac and did contract-based research for Virbac. The funders, however, had no influence on the results. If there are other authors, they declare that they have no known competing financial interests or personal relationships that could have appeared to influence the work reported in this paper.

## Data Availability

Data will be made available on request.
